# Flavonoid Inhibition of *Bacillus licheniformis* LP‐8 Lipase: In Vitro and In Silico Insights Into Anti‐Obesity Potential

**DOI:** 10.1002/open.70227

**Published:** 2026-05-14

**Authors:** Songul Bayrak, Mehmet Akif Omeroglu, Halil Senol

**Affiliations:** ^1^ Department of Chemistry Faculty of Science Ataturk University Erzurum Turkey; ^2^ Department of Molecular Biology and Genetics Faculty of Science Ataturk University Erzurum Turkey; ^3^ Department of Pharmaceutical Chemistry Faculty of Pharmacy Bezmialem Vakif University Istanbul Turkey

**Keywords:** *Bacillus licheniformis* LP‐8 lipase, enzyme inhibition, flavonoids, molecular docking

## Abstract

Lipase inhibition is a key strategy for controlling dietary fat absorption and managing obesity. In this study, lipase produced from *Bacillus licheniformis* LP‐8 (GenBank accession number: PX970421), using waste frying oil, was used to evaluate the inhibitory potential of selected flavonoid compounds. In vitro inhibition assays revealed IC_50_ values ranging from 1.28 to 3.51 μM, with syringetin exhibiting the strongest inhibitory activity (IC_50_ = 1.28 ± 0.009 μM), surpassing the reference inhibitor, orlistat (IC_50_ = 2.13 ± 0.010 μM). Structure–activity relationship analysis indicated that electron‐donating substituents, particularly methoxy and hydroxyl groups on the B‐ring, play a crucial role in enhancing lipase inhibition. To further elucidate the interaction mechanisms, molecular docking and molecular dynamics (MD) simulations were performed. Induced Fit Docking results demonstrated favorable binding affinities for syringetin, diosmin, and isorhamnetin‐3‐O‐rutinoside, with syringetin showing the most stable binding profile. Subsequent 250 ns MD simulations confirmed the structural stability of the lipase–syringetin complex through persistent hydrogen bonding and π–π interactions, indicating a well‐oriented and stable binding mode. Overall, the combined experimental and computational findings highlight the potential of flavonoids, particularly syringetin, as promising natural lipase inhibitors for obesity‐related applications.

## Graphical abstract

*Bacillus licheniformis* LP‐8, isolated from hot spring, produced lipase using waste frying oil. Flavonoids inhibited enzyme activity, confirmed by reduced activity assays. Molecular docking revealed hydrogen‐bond interactions within the lipase active site. The graphical abstract was created with the assistance of a generative artificial intelligence tool (ChatGPT).

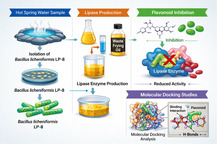

## 1 Introduction

Triglycerides (TG) are high‐energy‐density lipid components of food, and the overconsumption of TG is regarded as a major risk factor at the cellular level. TG are absorbed by the intestine, however, they are not absorbed directly, as they must first be hydrolyzed by lipases into free fatty acids, monoacylglycerols, or diacylglycerols prior to uptake by enterocytes [1, 2]. In this pathway, lipases are essential for the emulsification of dietary fats and for their metabolic availability.

Lipases (EC 3.1.1.3) are a widespread class of enzymes that catalyze the hydrolysis of triacylglycerols and can be obtained from various biological sources, including plants, animals, and microorganisms. Lipases of microbial origin are particularly useful in biotechnology and the pharmaceutical industry because of their efficient catalytic activity, broad substrate specificity, and good stability. Accordingly, microbial lipases have become ideal model enzymes for investigating lipid metabolism and developing new lipase inhibitors [3, 4]. Although pancreatic lipase is the primary pharmacological target for obesity management, microbial lipases are frequently employed as model systems in inhibitor screening studies due to their structural and mechanistic similarities. Both microbial and pancreatic lipases belong to the α/β‐hydrolase fold family and share a highly conserved catalytic triad (Ser–His–Asp), as well as comparable hydrophobic substrate‐binding pockets. In particular, lipases from Bacillus species have been reported to exhibit active site architectures and catalytic behaviors analogous to those of pancreatic lipases, making them suitable for preliminary mechanistic and structure–activity relationship investigations. In this context, thermophilic *Bacillus licheniformis* lipase provides a stable and well‐characterized model system for evaluating ligand–enzyme interactions under controlled experimental and computational conditions [5, 6].

Success in suppressing lipase activity has been regarded as an effective method to lower caloric intake by preventing fat hydrolysis [7]. The most popular lipase inhibitor in clinical use is orlistat, a partially synthetic derivative of the natural product lipstatin. Orlistat shows potent inhibition through covalent binding to the active site serine of lipases. However, excessive suppression of lipase activity may cause side effects such as steatorrhea, abdominal distension, and gastrointestinal disturbances. In this sense, it is clear that new, safer lipase inhibitors with controlled efficacy and enhanced individual tolerability are required [5, 8].

In this regard, phenolic compounds in foods have been considered good candidates for lipase inhibition, particularly flavonoids [9]. Flavonoids represent the most abundant subgroup of plant‐derived polyphenols and are characterized by a three‐ring structure consisting of two aromatic rings (A and B) and a heterocyclic C ring [10]. Structurally, flavonoids are divided into several categories, such as flavones, flavanones, isoflavones, flavonols, and flavan‐3‐ols [11].

It has been reported that flavonoids can inhibit several hydrolytic enzymes, particularly lipases, and that such inhibitory activity is largely structure‐dependent [6, 12, 13, 14]. In particular, numerous studies have demonstrated that flavonoids exhibit significant inhibitory activity against pancreatic lipase, including both porcine and human isoforms. For instance, quercetin, rutin, and related flavonoids have been reported to reduce lipid absorption by inhibiting lipases, highlighting their potential as natural anti‐obesity agents [4, 14, 15]. These findings provide a physiologically relevant context for evaluating flavonoid–lipase interactions. The existing literature has predominantly focused on pancreatic and fungal lipases, whereas lipases derived from *B. licheniformis* remain comparatively underexplored. However, microbial lipases such as those from *B. licheniformis* offer several notable experimental advantages, including high stability, ease of production, and suitability for detailed mechanistic investigations and structure–activity relationship analyses [16, 17].

**FIGURE 1:**
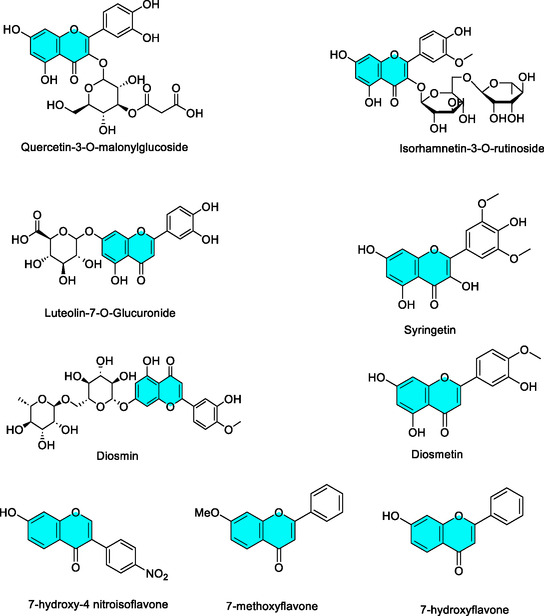
Chemical structures of the flavonoid compounds examined in this study.

In this study, *B. licheniformis* lipase was used as a model enzyme to investigate flavonoid interactions (a total of nine compounds, Figure 1) with lipases, aiming to provide detailed mechanistic insights into enzyme–inhibitor interactions that may contribute to the rational design of lipase inhibitors.

## 2 Materials and Methods

### 2.1 Materials

Reagents and chemicals: All solutions/reference materials were AR‐grade and used as received. Test inhibitors: The flavonoid standards were purchased from commercial suppliers with good standing and ≥97% purity, as reported by the manufacturer. The bacterial lipase used in the study was obtained from waste oil samples, partially purified, and used in an in vitro inhibitory assay. Lipase activity was measured using a substrate and buffers prepared in accordance with standard methods.

### 2.2 Isolation of Thermophilic Bacterial Isolates and Screening Their Lipolytic Abilities

Water samples from hot springs in Erzurum province, Turkey [Pasinler (39° 58′ 37″ N 41° 39′ 56″ E) and Ilica (39° 56′ 17″ N 41° 06′ 29″ E)] were used as the isolation source for lipase‐producing thermophilic bacteria. A volume of 100 μL from each water sample was inoculated into sterile flasks containing Tryptic Soy Broth (TSB). The flasks were then incubated at 55°C for 24–48 h in a shaking incubator to enrich the bacteria. After cultivation, the samples were serially diluted with physiological sterile water (0.9% NaCl) and spread on mineral salt medium (MSM) agar supplemented with 20 mL/L waste frying oil (pH 7.0). All agar plates were left to incubation at 40°C–60°C for 24–48 h and bacterial colonies exhibiting different morphological characteristics on the plates were selected, subcultured, and purified [18].

Screening experiments were performed in 250 mL flasks containing 100 mL of waste frying oil‐based fermentation medium (20 mL/L waste frying oil and MSM, pH 7.0). 1 mL (OD600 = 1.0) of preculture of the isolates was used for the inoculation of screening medium and inoculated flasks were then left to incubation at 55°C and 150 rpm. After 48 h of incubation, the fermentation broths were centrifuged at 10,000 rpm for 10 min, and the resulting cell‐free supernatant was used as enzyme source for the lipase activity assay. For this purpose, an aliquot of 0.2 mL of the supernatant was combined with 1 mL of 0.05 M phosphate buffer (pH 7.0) and 1 mL of substrate solution containing 0.013 M p‐nitrophenyl palmitate dissolved in ethanol. The reaction mixture was incubated at 55°C for 5 min and subsequently terminated by the addition of 2 mL of 0.5 M Na_2_CO_3_. The mixture was then centrifuged at 10,000 rpm for 10 min, and the absorbance of the resulting supernatant was measured at 410 nm using a Beckman Coulter DU730 spectrophotometer. One unit (U) of lipase activity was defined as the amount of enzyme required to release 1 µmol of p‐nitrophenol per minute under the assay conditions [19].

### 2.3 Molecular Identification of the Best Lipase‐Producing Isolate

Identification of the isolate at molecular level was performed by sequencing the 16S rRNA gene region. Genomic DNA of the strain was isolated according to the Promega WizardR Genomic DNA Purification Kit (A2360) protocol [20]. 16S rRNA gene region was then amplified using universal primers of 27F (5′‐AGAGTTTGATCCTGGCTCAG‐3′) and 1492R (5′‐GGTTACCTTGTTACGACTT‐3′). The amplified products were cloned into *Escherichia coli* JM101 strain with the pGEM‐T Easy Cloning Vector (Promega, Southampton, UK) in compliance with the instructions of the manufacturer. Afterwards, 16S rRNA gene region was sequenced in the Macrogen (Netherlands) and the obtained sequence was compared with the other bacterial series in GenBank and EzBioCloud (http://blast.ncbi.nlm.nih.gov and https://www.ezbiocloud.net). Similarity rate between them was determined and GenBank accession number was received. A phylogenetic tree was constructed with Mega4 software based on the 16S rRNA sequences [21].

### 2.4 Determination of Lipase Inhibitory Activity

The flavonoid compounds were analyzed for their inhibition of lipase activity by spectrophotometric analysis with p‐nitrophenyl palmitate (p‐NPP) as the substrate. Stock solution of flavonoids (at constant concentration) was serially diluted with a suitable solvent, and various volumes were added to the reaction mixture to obtain various final inhibitor concentrations. The volume of phosphate buffer was varied, as required, such that the total reaction volume was kept constant in all groups. The mixture solution was comprised of 0.2 mL of enzyme, which contains lipase; 0.05 M phosphate buffer (pH 7.0); flavonoid compounds in different concentrations, and extra phosphate buffer (0.05 M, pH 7) to make volume balance. Enzyme–inhibitor mixtures were preincubated at 55°C for 10 min. Then, 1.0 mL of 0.013 M p‐NPP (prepared in ethanol) was added to the reaction mixture and incubated for 5 min at 55°C. The reaction was stopped by adding 2.0 mL of 0.5 M Na_2_CO_3_. The mixtures were centrifuged at 10,000 rpm for 10 min, and the absorbance of the supernatant was determined at 410 nm using a Beckman Coulter DU730 spectrophotometer [5].

Flavonoid stock solutions were prepared in dimethyl sulfoxide (DMSO). The final DMSO concentration in the reaction mixture did not exceed 1% (v/v), and control experiments confirmed that this concentration had no significant effect on lipase activity. To exclude possible interference with spectrophotometric measurements, each flavonoid compound was tested under identical conditions in the absence of enzyme. No significant absorbance at 410 nm was observed.

#### 2.4.1 Determination of IC_50_ Values

The inhibitory activity of the flavonoid compounds on the lipase was determined by measuring the enzyme activity (%) in biochemical studies at different inhibitor concentrations. The enzyme activity was expressed as the percentage of absorbance recorded in the presence of each flavonoid concentration relative to that measured without the inhibitor, which was considered 100% activity. The remaining enzyme activity was expressed as a percentage. Subsequently, for each flavonoid compound, enzyme activity (%) was plotted against flavonoid concentration (µM), and IC_50_ values were determined from the corresponding inhibition curves. IC_50_ was defined as the concentration of flavonoid required to reduce lipase activity by 50% relative to the control [22, 23, 24].

### 2.5 Computational Studies

Computational studies were performed using the Schrödinger Maestro suite. Molecular docking calculations were carried out with Glide, and molecular dynamics (MD) simulations were conducted using Desmond implemented in Maestro. The crystal structure of thermophilic *B. licheniformis* lipase (PDB ID: 6WPY) was obtained from the Protein Data Bank and prepared using the Protein Preparation Wizard, including bond order assignment, addition of missing hydrogen atoms, optimization of protonation states, and restrained energy minimization with the OPLS4 force field. Potential ligand‐binding pockets were identified using SiteMap, and two binding sites were detected; based on pocket size and electrostatic characteristics, the larger and more favorable site was selected for subsequent docking studies. Ligands were prepared using LigPrep to generate optimized 3D structures and appropriate ionization states at physiological pH. The receptor grid was generated based on the selected SiteMap‐defined binding pocket. Docking with Glide XP was carried out, followed by IFD of the docked poses to consider receptor flexibility, and best ranked poses based on docking scores and interaction profiles were chosen. These resulting ligand–protein complexes were then subjected to 250 ns MD simulation in NPT (300 K, 1 atm) using the OPLS4 force field and a TIP4P water model. Trajectory analyses, including root mean square deviation (RMSD), root mean square fluctuation (RMSF), and fractional interaction histograms, were conducted to evaluate the conformational stability of the complexes and to identify key residues contributing to ligand binding throughout the simulation period [25, 26, 27, 28].

### 2.6 Statistical Analysis

All experiments were performed in triplicate for each sample. Results are presented as mean ± standard deviation (SD, *n* = 3). Statistical analyses were carried out using two‐way analysis of variance, followed by Tukey's post hoc test. Differences were considered statistically significant at *p* < 0.05.

## 3 Results and Discussion

### 3.1 Isolation and Screening of Thermophilic Bacteria From Hot Springs

**FIGURE 2:**
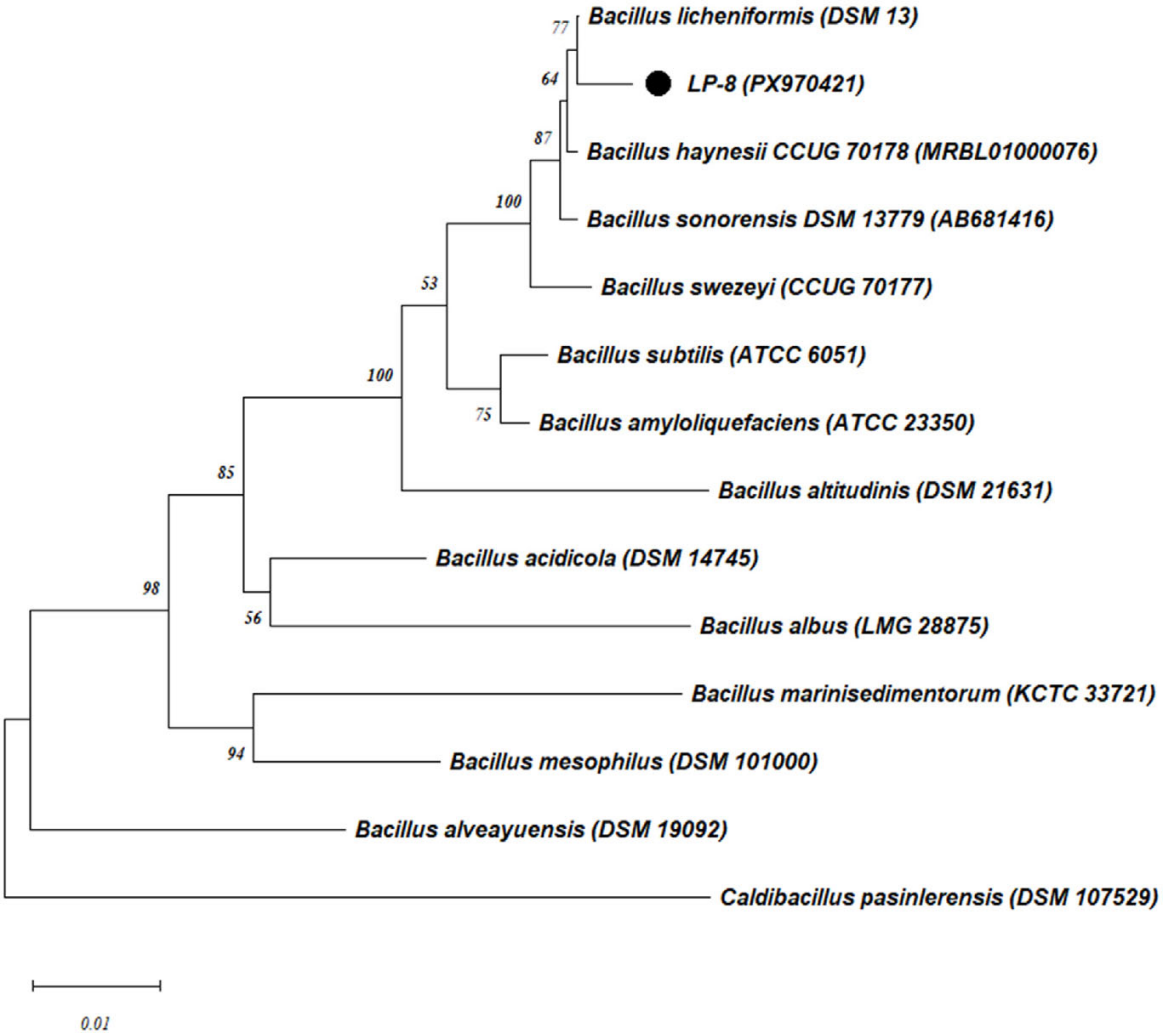
Phylogenetic tree of the isolate LP‐8 on the basis of 16S rRNA gene sequence. Tree was constructed by a neighbor‐joining method. Bootstrap values were based on 100 replicates. *Caldibacillus pasinlerensis* was used as the out‐group. The scale bar represents 0.01 changes per nucleotide position.

During the initial phase of the study, thermophilic bacteria were isolated from various hot spring samples, yielding a total of 11 strains grown on waste frying oil–containing agar media. The isolation medium was prepared using waste frying oil as the sole carbon source, without the addition of any other carbon sources. These isolates were subsequently assessed for their lipolytic activity. Screening experiments revealed that, when cultivated in liquid culture, the strain LP‐8 exhibited the highest lipase activity (650 U/L) with the specific activity of 6.5 U/mg. Consequently, the isolate LP‐8 was selected for further investigation in the subsequent stages of the study. Based on 16S rRNA gene sequence analysis, the isolate LP‐8 showed similarity to *B. licheniformis* with the rate of 99% (GenBank number: PX970421). The evolutionary relationship of WL5 was assessed using the neighbor‐joining method (Figure 2).

### 3.2 Lipase Enzyme Inhibition

Lipase is a principal digestive enzyme that hydrolyzes dietary TG and plays a critical role in regulating fat absorption and overall energy intake. Accordingly, inhibition of lipase activity is widely recognized as an effective strategy for managing obesity and related metabolic disorders [15, 29]. It should be noted that all inhibition assays were conducted at 55°C, corresponding to the optimal activity of the thermophilic lipase used in this study. Although orlistat is typically evaluated at physiological temperature (37°C), additional control experiments confirmed that its inhibitory profile remained consistent across both temperature conditions, indicating that the elevated assay temperature did not bias the comparative analysis.

**FIGURE 3:**
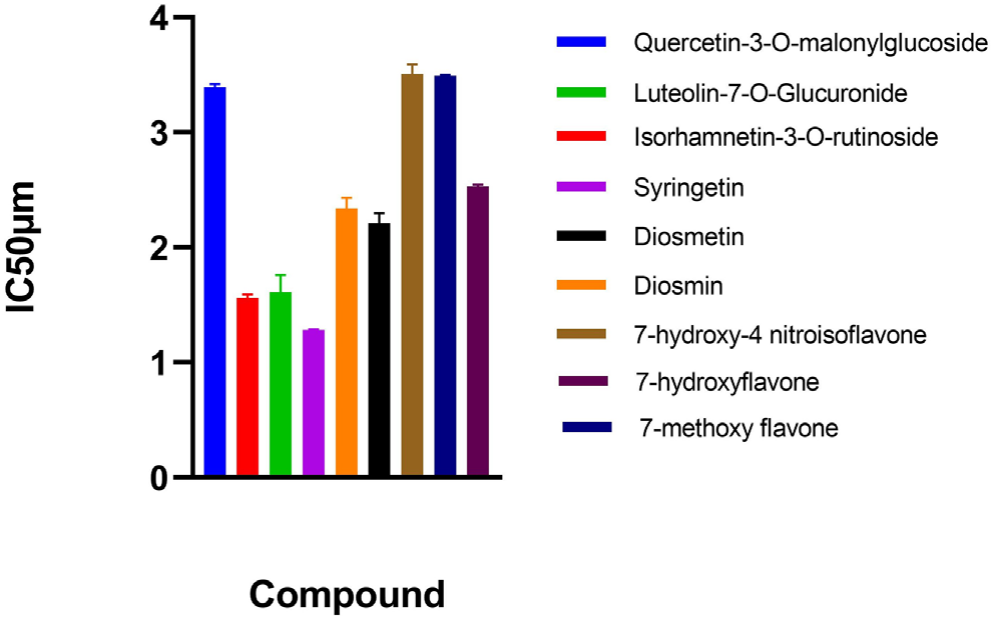
Comparative IC_50_ values of flavonoids and the reference inhibitor orlistat against bacterial lipase.

All tested flavonoid compounds exhibited dose‐dependent inhibition of lipase activity, and post hoc multiple comparison analysis (Tukey's test) revealed that syringetin (1.28 µM), isorhamnetin‐3‐O‐rutinoside (1.56 µM), and luteolin‐7‐O‐glucuronide (1.61 µM) differed significantly from orlistat (2.13 µM) (*p* < 0.05), whereas diosmetin (2.21 µM) and diosmin (2.21 µM) showed no statistically significant difference compared to the reference inhibitor. Additionally, less active compounds such as quercetin‐3‐O‐malonylglucoside and methoxy‐substituted flavones formed distinct groups with significantly higher IC_50_ values (Table 1, Figure 3). These findings highlight the strong inhibitory capacity of flavonoids and support their potential as natural lipase inhibitors with promising bioactivity profiles.

**TABLE 1:** Inhibitory effects of selected flavonoid compounds on bacterial lipase activity expressed as IC_50_ values (mean ± SD, *n* = 3).

Compounds	**IC**_**50**_ **(µm)**	***R*** ^**2**^
Quercetin‐3‐O‐malonylglucoside	3.39 ± 0.030^d^	0.935
Isorhamnetin‐3‐O‐rutinoside	1.56 ± 0.030^a^	0.983
Luteolin‐7‐O‐Glucuronide	1.61 ± 0.150^a^	0.912
Syringetin	1.28 ± 0.009^a^	0.902
Diosmin	2.34 ± 0.092^b^	0.888
Diosmetin	2.21 ± 0.090^b^	0.940
7‐hydroxy‐4‐nitroisoflavone	3.51 ± 0.080^d^	0.979
7‐methoxy flavone	3.49 ± 0.010^d^	0.919
7‐hydroxyflavone	2.53 ± 0.015^c^	0.973
Orlistat	2.13 ± 0.010^b^	0.981

Different letters (a–d) indicate statistically significant differences between groups according to Tukey's post hoc test (*p* < 0.05).

The inhibitory activity of flavonoids against lipase was found to be strongly dependent on their molecular structures. Specifically, the degree of hydroxylation, methoxylation pattern, presence of glycosylation, and the nature and position of substituents on the phenyl (B) ring were identified as key determinants of inhibitory potency. Electron‐donating groups, such as hydroxyl (–OH) and methoxy (–OCH_3_) substituents, particularly when located on the phenyl ring, increase the π‐electron density of the aromatic system. This enhancement facilitates stronger interactions with hydrophobic and aromatic residues within the lipase binding pocket, thereby improving inhibitory efficiency [30, 31, 32].

The pronounced inhibitory potency of syringetin can be attributed to the presence of methoxy groups at the 3′ and 5′ positions of the phenyl ring, which enhance lipophilic interactions, together with multiple hydroxyl groups capable of forming hydrogen bonds with amino acid residues in the lipase active site, particularly those constituting the Ser–His–Asp catalytic triad. It has been reported that electron‐donating substituents at the para or meta positions on the phenyl ring increase the stability of the enzyme–inhibitor complex, thereby resulting in lower IC_50_ values [6, 33].

Among the glycosylated flavonoids, isorhamnetin‐3‐O‐rutinoside and luteolin‐7‐O‐glucuronide demonstrated notably strong inhibitory activities. The attached sugar moieties are likely to enhance the stability of the enzyme inhibitor complex by forming additional hydrogen bonds with polar regions on the lipase surface. Nevertheless, the influence of glycosylation on inhibitory activity appears to be highly dependent on both the type of sugar and its attachment position on the flavonoid backbone [34, 35, 36]. A comparison between diosmetin and its glycosylated counterpart, diosmin, revealed that diosmetin exhibited a lower IC_50_ value, suggesting that free hydroxyl groups are more favorable for lipase inhibition than glycosylated structures. This observation is consistent with previous reports indicating that the sugar moiety in diosmin may partially hinder access to the enzyme active site by introducing steric constraints [37]. Similarly, the significantly greater inhibitory activity of 7‐hydroxyflavone than that of 7‐methoxyflavone (*p* < 0.05) further underscores the critical contribution of hydroxyl groups to lipase inhibition. Based on the IC_50_ values obtained, the overall lipase inhibitory activity of the tested flavonoids was ranked as follows:

Syringetin > Isorhamnetin‐3‐O‐rutinoside ≈ Luteolin‐7‐O‐glucuronide > Diosmetin ≈ Diosmin ≈ Orlistat > 7‐hydroxyflavone > Quercetin‐3‐O‐malonylglucoside > 7‐methoxyflavone ≈ 7‐hydroxy‐4‐nitroisoflavone (Table 2, Figure 3).

**TABLE 2:** IFD scores and MMGBSA Δ*G* binding free energies of title compounds.

Compounds	IFD docking score (kcal/mol)	MMGBSA Δ*G* Bind (kcal/mol)
Quercetin‐3‐*O*‐malonylglucoside	−10.223	−65.00
Isorhamnetin‐3‐*O*‐rutinoside	−11.213	−63.38
Diosmin	−11.109	−68.55
Luteolin‐7‐*O*‐Glucuronide	−9.703	−42.21
Diosmetin	−7.779	−45.20
Syringetin	−11.776	−68.10
7‐hydroxy‐4‐nitroisoflavone	−8.370	−55.46
7‐methoxyflavone	−8.196	−49.27
7‐hydroxyflavone	−8.043	−49.97

The inhibitory potency of the tested flavonoids was found to be comparable to or higher than that reported in previous studies on lipase inhibition. It has been shown that many flavonoids exhibit inhibitory activity in the low‐ to moderate‐micromolar range, depending on their structural features and assay conditions. In particular, compounds such as quercetin, luteolin, and their derivatives have been reported to inhibit lipases, with IC_50_ values generally ranging from low‐ to high‐micromolar concentrations. In this context, the strong inhibitory activity of syringetin (IC_50_ = 1.28 μM), which exceeds that of orlistat under the present experimental conditions, highlights its potential as a promising lead compound. However, variations in enzyme source, substrate type, and assay methodology may influence IC_50_ values; therefore, direct comparisons between studies should be interpreted with caution [4, 6, 14, 34, 37].

### 3.3 Molecular Docking Studies

In the present study, the crystal structure of thermophilic *B. licheniformis* lipase was retrieved from the Protein Data Bank (PDB ID: 6WPY) and prepared prior to docking calculations. To identify the most suitable ligand‐binding region, SiteMap analysis was performed on the protein structure. As a result, two potential binding pockets were detected: one pocket was relatively small and limited in volume, whereas the second pocket exhibited a larger cavity size and higher SiteScore/ESP characteristics, indicating a more favorable environment for ligand accommodation. Therefore, subsequent docking simulations were carried out using the larger and more druggable binding site.

The amino acid residues constituting the selected binding region were identified as His‐54, Gly‐55, Leu‐56, Asn‐57, Gly‐58, Ser‐59, Ala‐62, Trp‐63, Glu‐66, Asn‐67, Tyr‐118, Ile‐168, Ile‐169, Phe‐255, Ile‐258, and Lys‐259. Importantly, this region was found to be located in close proximity to the catalytic active site of the enzyme. The catalytic triad residues (Ser–His–Asp), which are essential for lipase activity, are positioned near this binding pocket, along with residues contributing to substrate stabilization. These findings suggest that the selected binding site is functionally associated with the catalytic region of the enzyme. Therefore, the binding of flavonoids within this pocket may interfere with substrate access and catalysis, indicating a potential competitive type of inhibition mechanism.

To explore the binding potential of phytochemical ligands, a set of selected natural products was docked into the identified binding pocket. The evaluated compounds included Quercetin‐3‐O‐malonylglucoside, Isorhamnetin‐3‐O‐rutinoside, Diosmin, Luteolin‐7‐O‐glucuronide, Diosmetin, Syringetin, 7‐hydroxy‐4‐nitroisoflavone, 7‐methoxyflavone, and 7‐hydroxyflavone. Docking calculations were performed using the Induced Fit Docking (IFD) protocol to account for receptor flexibility and obtain reliable ligand–protein interaction conformations. Following docking, the binding affinities of the best‐ranked complexes were further validated by calculating the binding free energies using the MM‐GBSA approach. The detailed docking scores and MM‐GBSA binding energies are summarized in Table 2.

Table 2 summarizes the IFD docking scores and MM‐GBSA binding free energies (Δ*G* bind) of the selected natural products against *B. licheniformis* lipase (PDB: 6WPY). Overall, the compounds exhibited favorable binding tendencies within the selected large binding pocket, with IFD scores ranging from −7.779 to −11.776 kcal/mol and MM‐GBSA Δ*G* bind values between −42.21 and −68.55 kcal/mol. Among the tested ligands, Syringetin, Diosmin, and Isorhamnetin‐3‐O‐rutinoside showed the most promising computational binding profiles, yielding the lowest docking scores and highly negative Δ*G* bind values, suggesting strong and stable interactions with the active site region.

Interestingly, Luteolin‐7‐O‐glucuronide despite displaying a comparatively weaker predicted binding affinity (IFD = −9.703 kcal/mol; Δ*G* bind = −42.21 kcal/mol), was identified as the most active compound in vitro. In the case of luteolin‐7‐O‐glucuronide, the discrepancy between its strong in vitro inhibition and relatively weaker in silico binding scores may be attributed to differences in binding modes and the dynamic nature of enzyme–ligand interactions, which are not fully captured by docking approaches. Similar inconsistencies between experimental and computational results have been reported in previous studies, where factors such as ligand flexibility, solvent effects, and alternative binding conformations influence inhibitory activity. In particular, glycosylated flavonoids may exhibit additional interactions in the experimental system that are not fully represented in docking calculations.

**FIGURE 4:**
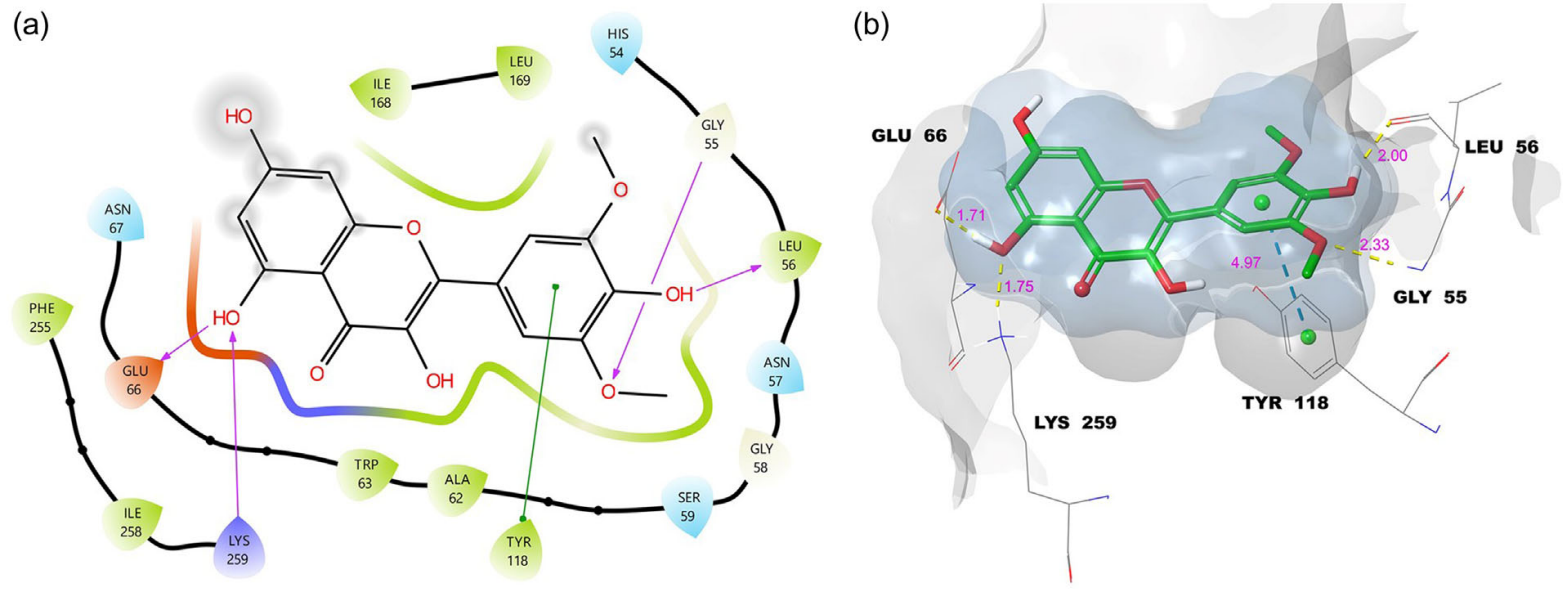
Molecular docking 2D (a) and 3D (b) ligand protein interactions between syringetin and the active site of lipase.

Therefore, a detailed pose analysis was performed for syringetin, and its 2D/3D interaction maps are presented in Figure 4.

As shown in Figure 4a, the phenolic hydroxyl groups of syringetin formed a total of four hydrogen bonds with Leu‐56, Glu‐66, and Lys‐259, while the methoxy oxygen additionally interacted with Gly‐55 via hydrogen bonding. Moreover, the B‐ring of syringetin established a π–π stacking interaction with Tyr‐118. These multiple hydrogen bonds, together with the supportive π–π stacking contact, are crucial for stabilizing the ligand–protein complex. Figure 4b illustrates the 3D LPI representation, where yellow dashed lines indicate hydrogen bonds and turquoise dashed lines represent π–π stacking interactions. The gray surface corresponds to the protein binding surface area, whereas the blue surface represents the ligand surface. Notably, the surfaces show an almost complete overlap (~100%), confirming the excellent orientation and fitting of syringetin within the binding cavity. The hydrogen bond distances ranged from 1.70 to 2.33 Å, while the π–π stacking distance was calculated as 4.97 Å.

### 3.4 Molecular Dynamics Simulation

**FIGURE 5:**
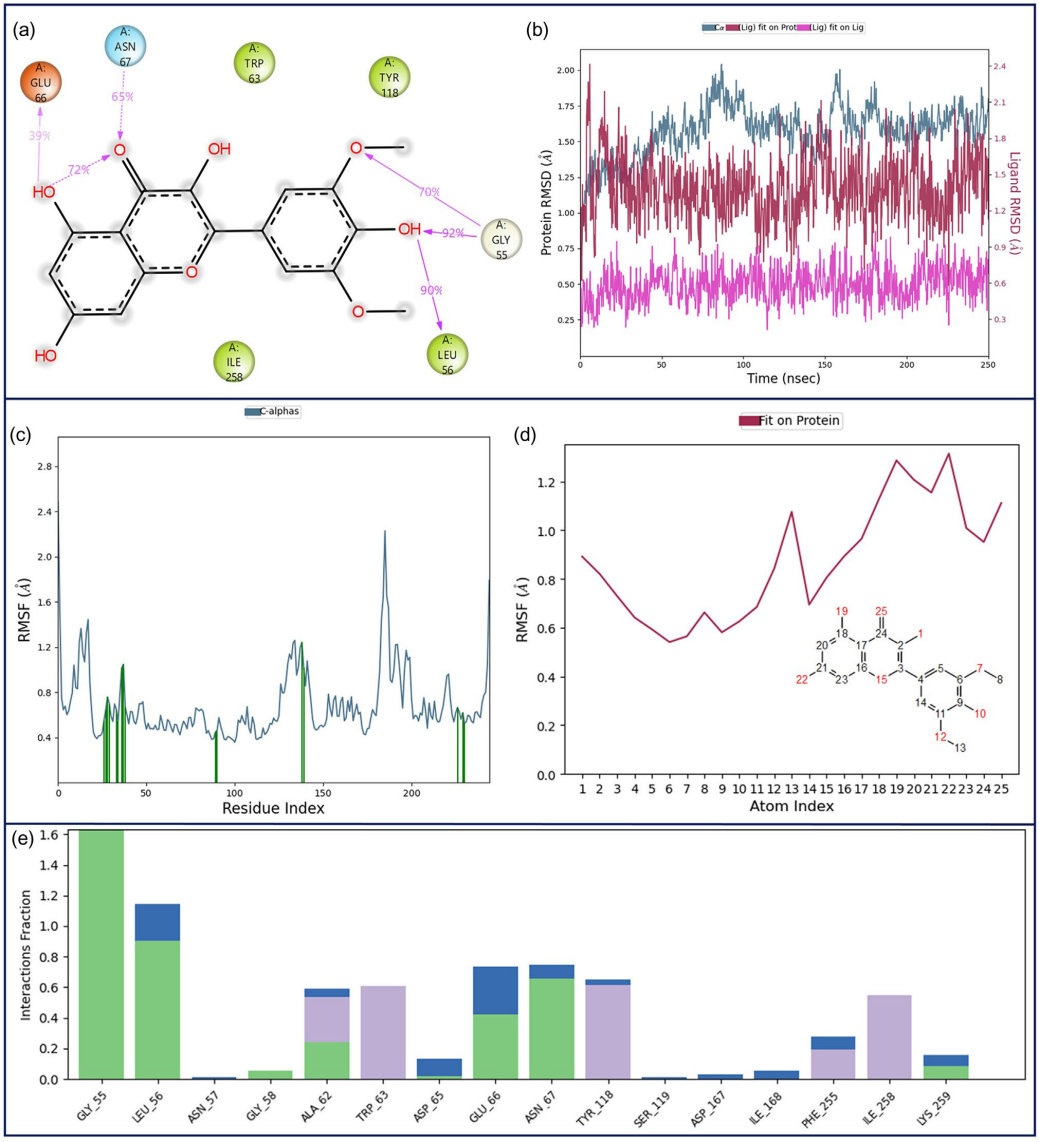
MD simulation analysis of syringetin–lipase complex. (a) 2D key LPI during the simulation time, (b) RMSD of protein and ligand atoms, (c) RMSF of protein atoms, (d) RMSF of ligand atoms, and (e) fractional interaction histogram.

To further validate the docking results and investigate the time‐dependent stability of the lipase–syringetin complex, a 250 ns MD simulation was performed. The simulation trajectories were evaluated using RMSD analysis to monitor the overall structural stability of the complex and the persistence of ligand binding throughout the run. In addition, RMSF analysis was carried out to assess residue‐level flexibility and to identify regions showing higher mobility during the simulation. A 250 ns MD simulation results are given in Figure 5.

Figure 5a represents the key 2D ligand–protein interaction profile of syringetin within the lipase binding pocket during the 250 ns MD simulation. An intramolecular hydrogen bond in the chromene ring between the carbonyl oxygen and hydroxyl group was maintained for 72% of the simulation time. In addition, the carbonyl oxygen formed a hydrogen bond with Asn‐67 with 65% occupancy. The phenolic hydroxyl groups established persistent hydrogen bonds with Glu‐66 (39%), Gly‐55 (92%), and Leu‐56 (90%), while Gly‐55 also interacted with the methoxy ether oxygen via hydrogen bonding for 70% of the simulation.

Figure 5b indicates the RMSD profiles of the protein and ligand atoms throughout the simulation. The average protein Cα RMSD was ≈ .50 Å (pale blue), indicating a stable protein backbone. The average ligand RMSD (fit on protein) was around 1.50 Å (red), suggesting that syringetin remained consistently positioned in the binding pocket, whereas the average ligand RMSD (fit on ligand) was 0.60 Å (pink), reflecting low conformational drift of the ligand.

Figure 5c, d reveals the RMSF profiles of the protein and ligand atoms, respectively. The average protein Cα RMSF was ≈ 1.0 Å, demonstrating limited residue‐level flexibility and overall structural stability of the lipase. Similarly, the average ligand RMSF was also around 1.0 Å, indicating that syringetin maintained a stable conformation while remaining bound within the active site throughout the simulation.

Figure 5e depicts the fractional interaction histogram summarizing the interaction frequencies between syringetin and lipase residues during the simulation. Since one residue may interact with multiple ligand functional groups (and vice versa), partial interaction contributions are accumulated to generate the total histogram. In this plot, green indicates hydrogen bonds, blue corresponds to water‐bridged hydrogen bonds, and purple represents hydrophobic interactions.

Direct hydrogen bonds and hydrophobic interactions play a primary role in stabilizing the ligand within the binding pocket and maintaining its proper orientation throughout the simulation. In addition, water‐bridged hydrogen bonds may further contribute to the stabilization of the ligand–protein complex by mediating indirect interactions between the ligand and surrounding residues, thereby enhancing the persistence of binding during the simulation [27, 38, 39]. The most dominant interacting residues were Gly‐55 and Leu‐56, while other notable contributors included Ala‐62, Trp‐63, Glu‐66, Asn‐67, Tyr‐118, and Ile‐258.

## 4 Conclusion

This study shows that flavonoids are effective inhibitors of bacterial lipase, underscoring their potential as natural compounds for the development synthetic inhibitors. The most effective compound was syringetin, which inhibited the enzyme more potently than the reference inhibitor drug orlistat, indicating the pivotal role of methoxy and hydroxyl substituents in the flavonoid skeleton. Docking and MM‐GBSA analyses were also consistent with experimental results, showing strong binding of syringetin within the druggable lipase pocket, mediated by multiple hydrogen bonds and π–π interactions with certain crucial active‐site residues. Moreover, 250 ns MD simulations also showed that the lipase–syringetin complex was stable. In summary, this combined in vitro and in silico study provides mechanistic insight into flavonoid lipase inhibition, and syringetin can be considered an effective lead compound for the development of a natural plant‐derived anti‐obesity agent.

## Conflicts of Interest

The authors declares no conflicts of interest.

## Data Availability

The data that support the findings of this study are available from the corresponding author upon reasonable request.
